# Clinical Significance of *Mycobacterium kansasii* Isolates from Respiratory Specimens

**DOI:** 10.1371/journal.pone.0139621

**Published:** 2015-10-02

**Authors:** Seong Mi Moon, Hye Yun Park, Kyeongman Jeon, Su-Young Kim, Myung Jin Chung, Hee Jae Huh, Chang-Seok Ki, Nam Yong Lee, Sung Jae Shin, Won-Jung Koh

**Affiliations:** 1 Division of Pulmonary and Critical Care Medicine, Department of Medicine, Samsung Medical Center, Sungkyunkwan University School of Medicine, Seoul, South Korea; 2 Department of Radiology, Samsung Medical Center, Sungkyunkwan University School of Medicine, Seoul, South Korea; 3 Department of Laboratory Medicine, Samsung Medical Center, Sungkyunkwan University School of Medicine, Seoul, South Korea; 4 Department of Microbiology, Yonsei University College of Medicine, Seoul, South Korea; Chang-Gung University, TAIWAN

## Abstract

The clinical significance of *Mycobacterium kansasii* respiratory isolates is uncertain. The aims of this study were to determine the clinical relevance of *M*. *kansasii* isolates and to identify the clinical features and outcomes of *M*. *kansasii* lung disease. We reviewed the medical records of 104 patients from whom at least one respiratory *M*. *kansasii* isolate was obtained from January 2003 to July 2014 at Samsung Medical Center, South Korea. Of these 104 patients, 54 (52%) met the diagnostic criteria for nontuberculous mycobacterial lung disease; among them, 41 (76%) patients received antibiotic treatment for a median time of 15.0 months (interquartile range [IQR], 7.0–18.0 months). The remaining 13 (24%) without overt disease progression were observed for a median period of 24.0 months (IQR, 5.0–34.5 months). Patients with *M*. *kansasii* lung disease exhibited various radiographic findings of lung disease, including the fibrocavitary form (n = 24, 44%), the nodular bronchiectatic form (n = 17, 32%), and an unclassifiable form (n = 13, 24%). The fibrocavitary form was more common in patients who received treatment (n = 23, 56%), while the nodular bronchiectatic form was more common in patients with *M*. *kansasii* lung disease who did not receive treatment (n = 9, 70%). None of the patients with a single sputum isolate (n = 18) developed *M*. *kansasii* disease over a median follow-up period of 12.0 months (IQR, 4.0–26.5 months). In total, 52% of all patients with *M*. *kansasii* respiratory isolates exhibited clinically significant disease. Moreover, patients with *M*. *kansasii* lung disease displayed diverse radiographic findings in addition to the fibrocavitary form. The nodular bronchiectatic form was more common in patients with *M*. *kansasii* lung disease with an indolent clinical course. Thus, since the clinical significance of a single *M*. *kansasii* respiratory isolate is not definite, strict adherence to recommended diagnostic criteria is advised.

## Introduction

Nontuberculous mycobacteria (NTM) are environmental organisms that can be isolated from water, soil, plants, animals, and dust [1, 2]. NTM are increasingly recognized as pathogenic to humans, with pulmonary disease being the most common manifestation of NTM infection [3, 4]. Unlike *Mycobacterium tuberculosis*, NTM derived from respiratory isolates must be examined to distinguish colonizer or contaminant from a true pathogen responsible for lung disease. Therefore, the American Thoracic Society (ATS) and the Infectious Diseases Society of America (IDSA) have issued diagnostic criteria for NTM lung disease [5].

*Mycobacterium kansasii* is a slow-growing NTM and the second most common cause of NTM lung disease in some European countries including the United Kingdom, Slovakia, and Poland [6]. Traditionally, *M*. *kansasii* has been considered the most virulent NTM species and the presence of a single *M*. *kansasii* isolate in a sputum sample has been believed to be clinically significant by many experts [7, 8]. However, the degree of correlation between *M*. *kansasii* isolation respiratory samples and true lung disease has not been definitely established. Previous reports from the United States and the United Kingdom found that more than 70% of all patients with a respiratory isolate of *M*. *kansasii* had clinically relevant disease [9–11]. However, studies from Asian countries found that only 17% of all *M*. *kansasii* isolates from respiratory samples were clinically relevant according to the ATS/IDSA diagnostic criteria [12].

The typically recognized clinical presentation of NTM lung disease, including *M*. *kansasii* lung disease, is an apical fibrocavitary form that is nearly identical to pulmonary tuberculosis. This type of disease usually develops in older males with a history of lung disease, such as previous pulmonary tuberculosis [5]. NTM lung disease can also present with nodular infiltrates, which frequently involve the right middle lobe and the lingular segment of the left upper lobe (nodular bronchiectatic form). This form of disease occurs predominantly in postmenopausal, non-smoking females and is also common in *Mycobacterium avium* complex (MAC) lung disease [13–15]. However, the nodular bronchiectatic form of *M*. *kansasii* lung disease has not been well described in the literature.

In South Korea, the frequency of NTM isolation and the number of patients diagnosed with NTM lung disease are both steadily increasing [16, 17]. *M*. *kansasii* has been shown to represent only 1–2% of all NTM isolates and pathogens in NTM lung disease in South Korea [16–18]. In this study, we reviewed the medical records of all patients from whom *M*. *kansasii* was isolated from respiratory specimens taken during a 12-year period at our institution. The aims of this study were to determine the clinical relevance of *M*. *kansasii* isolates and to identify the clinical features and outcomes of *M*. *kansasii* lung disease.

## Patients and Methods

Using our mycobacterial laboratory database, all patients for whom an isolate of *M*. *kansasii* was present in at least one of two or more respiratory specimens taken at Samsung Medical Center (a 1,961-bed referral hospital in Seoul, South Korea) between January 2003 and July 2014 were identified. This retrospective observational study was approved by the Institutional Review Board of Samsung Medical Center (IRB No. 2014-10-093) and full permission was granted to review and publish information obtained from patient records. Informed consent was waived for the use of patient medical data because patient information was anonymized and de-identified prior to analysis.

During the study period, NTM species were identified using polymerase chain reaction (PCR)-restriction fragment length polymorphism analysis or a PCR-reverse blot hybridization assay of the mycobacterial *rpoB* gene [19, 20]. Antimicrobial susceptibility testing was performed at the Korean Institute of Tuberculosis. The minimum inhibitory concentration (MIC) of rifampin and clarithromycin was evaluated via the broth microdilution method and interpreted according to the National Committee for Clinical Laboratory Standard guidelines [21], which had been implemented since January 2009 in Korea.

For each patient, clinical, microbiological and radiographic data were evaluated. Chest radiography scans and chest high-resolution computed tomography (HRCT) images were available for all patients and these images were reviewed by two of the authors (S. M. Moon and H. Y. Park) with respect to the presence or absence of nodules, consolidation, cavitary lesions, and bronchiectasis [22]. Patients were then diagnosed with *M*. *kansasii* lung disease if they met the 2007 ATS/IDSA diagnostic criteria [5]. After evaluating the clinical significance the *M*. *kansasii* isolates, the chest radiography and HRCT findings were classified as showing either the fibrocavitary form or the nodular bronchiectatic form. If the disease did not belong to either of these categories, it was deemed unclassifiable [23, 24].

In patients with extensive lesions and respiratory symptoms, physicians started treatment for *M*. *kansasii* lung disease immediately. However, if patients had mild symptoms without clear progression, treatment was not initiated and they were instead followed regularly and sputum cultures were collected. During the follow-up period, patients who showed disease progression began treatment. At that time, the goal of treatment was maintenance of negative sputum conversion over 12 months with improvement in symptoms and performed images [15, 23]. When the patients were treated for *M*. *kansasii* lung disease, sputum conversion was defined as three consecutive negative cultures, with the time of conversion defined as the date of the first negative culture [15, 23]. The patients who completed treatment were followed regularly including repeated sputum acid-fast bacilli (AFB) culture for investigation of recurrence. Microbiologic recurrence was defined as two consecutive positive cultures after sputum conversion. Treatment outcomes were evaluated on December 31, 2014. At this point, if the intended treatment was finished, patient were categorized as having completed treatment and if patients were receiving treatment, they were categorized in the ongoing treatment group. If antibiotics were stopped with or without follow-up, patients were categorized as ‘discontinued’ or ‘lost to follow-up,’ respectively. Transfer of the patient to another hospital was described as ‘transfer’; if the patient died in our hospital because of the progression of *M*. *kansasii* lung disease, they were classified as ‘death during treatment’. In addition, for each patient, the test result of anti-human immunodeficiency virus (HIV) antibody screening was reviewed retrospectively.

All data are presented as medians and interquartile ranges (IQRs) for continuous variables and as numbers (percentages) for categorical variables. Data were compared using the Mann-Whitney *U* test for continuous variables and Pearson’s χ^2^ test or Fisher’s exact test for categorical variables. All statistical analyses were performed using PASW Statistics 21 (SPSS Inc., Chicago, IL, USA).

## Results

### Baseline Characteristics

From January 2003 to July 2014, 104 patients were identified with *M*. *kansasii*-positive cultures from 230 respiratory specimens. These specimens consisted of 217 sputum samples, 12 bronchial washing fluid samples and 1 lung tissue aspiration sample. Out of 104 patients, 102 patients had three or more available specimens. The patient baseline characteristics are detailed in Table 1. Of the 104 patients with *M*. *kansasii* respiratory isolates, 54 (52%) met the ATS/IDSA diagnostic criteria for NTM lung disease and 50 (48%) did not meet the 2007 ATS/IDSA criteria for *M*. *kansasii* lung disease. However, there was no significant difference in baseline characteristics between two groups. In total, 71 males (68%) and 33 females (32%) were included in this study, with a median age of 59 years (IQR, 45–67 years). The median body mass index was 20.6 kg/m^2^ (IQR, 18.8–22.9 kg/m^2^) and 48 (46%) patients were nonsmokers. The most frequent pre-existing pulmonary disease was a prior history of pulmonary tuberculosis (n = 39, 38%), followed by bronchiectasis (n = 37, 37%) and chronic obstructive pulmonary disease (n = 17, 16%). All patients were immunocompetent except one patient with myelodysplastic syndrome; Anti-HIV antibody test screening was performed in 85 (82%) of 104 patients, and none of the untested patients had risk factors for HIV infection.

**Table 1:** Baseline characteristics of the 104 patients with *M*. *kansasii*-positive cultures from respiratory specimens.

Characteristics	Total (n = 104)	Met ATS criteria (n = 54)	Did not meet ATS criteria (n = 50)	*P* value
Males, n (%)	71 (68)	39 (72)	32 (64)	0.368
Age, years	59 (45–67)	62 (45–66)	57 (42–67)	0.427
Body mass index, kg/m^2^	20.6 (18.8–22.9)	20.7 (18.7–22.8)	20.3 (18.9–23.0)	0.735
Current or ex-smoker, n (%)	60 (58)	35 (65)	25 (50)	0.127
Pre-existing pulmonary disease, n (%)				
Prior pulmonary tuberculosis	39 (38)	16 (30)	23 (46)	0.085
Bronchiectasis	38 (37)	15 (28)	23 (46)	0.054
Chronic obstructive pulmonary disease	17 (16)	11 (20)	6 (12)	0.249
Interstitial lung disease	6 (6)	4 (7)	2 (4)	0.680
History of previous lung surgery	8 (8)	3 (6)	5 (10)	0.477
Comorbidity, n (%)				
Malignancy	11 (11)	5 (9)	6 (12)	0.650
Diabetes mellitus	11 (11)	7 (13)	4 (8)	0.411
Immunocompromised disease*	1 (1)	1 (2)	0 (0)	1.000

All data are presented as numbers (%) or as medians and interquartile ranges.

ATS: 2007 American Thoracic Society (ATS) and the Infectious Diseases Society of America (IDSA) diagnostic criteria for nontuberculous mycobacterial lung disease.

*This patient had myelodysplastic syndrome.

All 104 patients with *M*. *kansasii* isolates were followed for a median of 21.7 months (IQR, 9.0–33.2 months). After *M*. *kansasii* was isolated, a median of 7.0 (IQR 3.0–11.0) sputum cultures were obtained during follow-up period.

### Comparison of Clinical Manifestations and Radiographic Findings Between Patients with *M*. *kansasii* Lung Disease Who Received Antibiotic Treatment Versus Those Who Did Not

**Fig 1:**
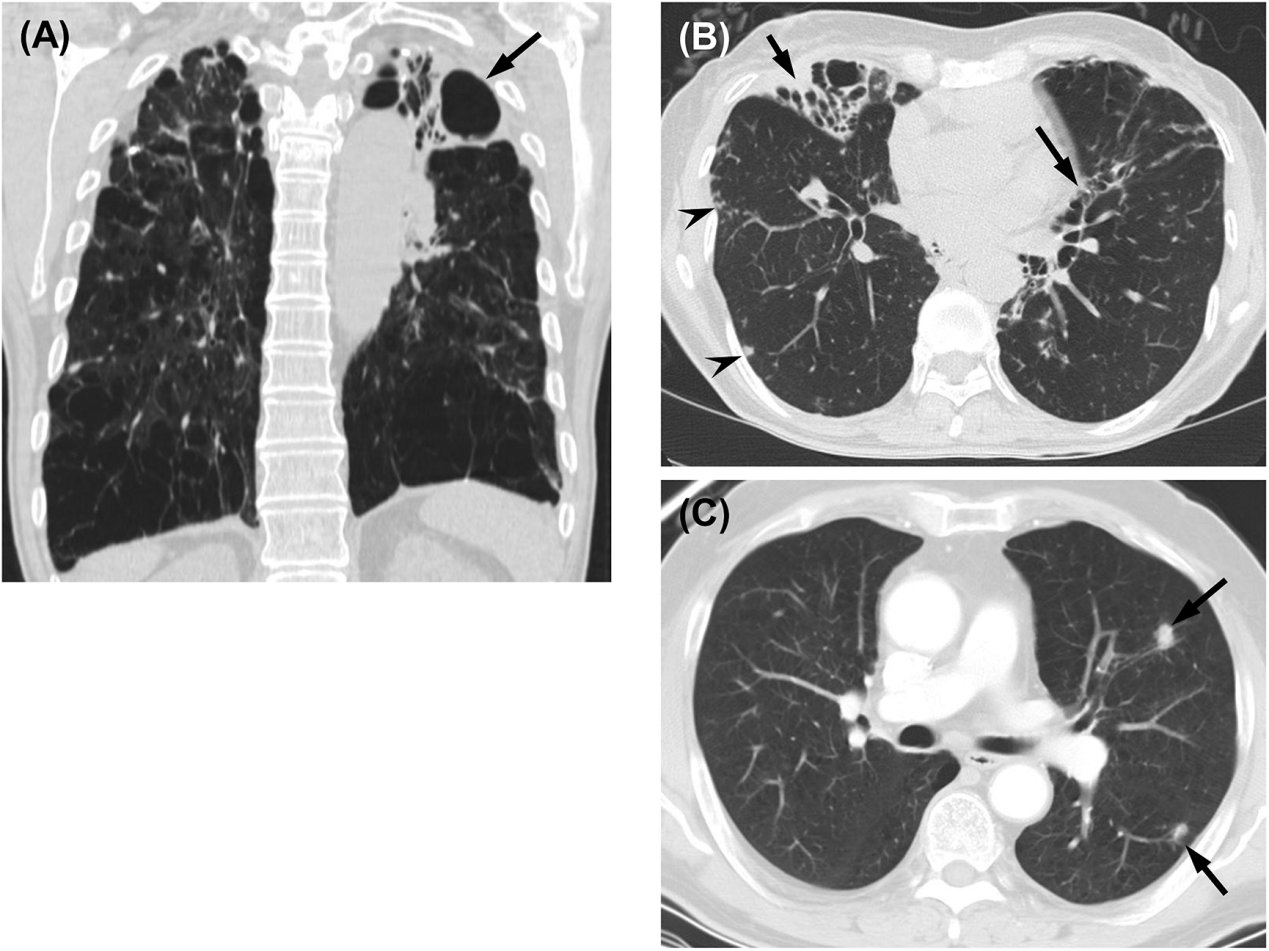
Diverse patterns in images of *M*. *kansasii* lung disease. (A) A 64-year-old man with *M*. *kansasii* lung disease. A coronal thin-section (2.5-mm thickness) CT scan shows a thick-walled cavitary lesion (arrow) with consolidation in the left upper lobe. Severe pulmonary emphysema is also observed. (B) A 66-year-old woman with *M*. *kansasii* lung disease. A transaxial thin-section (2.5-mm thickness) CT scan obtained at the basal trunk level shows bronchiectasis (arrows) and multiple branching centrilobular nodules, the so-called tree-in-bud pattern (arrowheads), in both lungs. (C) A-77-year-old man with *M*. *kansasii* lung disease. A transaxial (5-mm thickness) CT scan obtained at the level of the left main bronchus shows two nodules in the left lung (arrows). Lung biopsy revealed chronic granuloma; *M*. *kansasii* was isolated from the bronchial washing fluid.

Among 54 patients who met the ATS/IDSA diagnostic criteria for NTM lung disease, 41 (76%) received antibiotic treatment and the median duration from diagnosis to initiation of treatment was 1.7 months (0.3–3.8 months). The remaining13 (24%) patients with mild symptoms underwent regular follow-up without antibiotic treatment for a median time of 26.5 months (IQR, 8.8–35.3 months) and a median of 8.0 (IQR 3.0–11.5) sputum cultures were obtained during follow-up. Based on the chest radiography and HRCT findings in all 54 patients, 24 (44%) patients manifested the fibrocavitary form, 17 (32%) had the nodular bronchiectatic form, and 13 (24%) exhibited an unclassifiable form of *M*. *kansasii* lung disease. The diverse radiographic findings are shown in Fig 1.

We compared clinical and radiographic manifestations between patients with *M*. *kansasii* lung disease who received antibiotic treatment versus those who did not. As shown in Table 2, many of the clinical manifestations, including symptoms and the number of patients with a positive AFB smear, did not differ between the two groups. However, patients with *M*. *kansasii* lung disease who received antibiotic treatment were more likely to have the fibrocavitary form of disease than those who did not receive antibiotic treatment (56% vs. 8%, *p* = 0.002), while the nodular bronchiectatic form was observed more often in patients with *M*. *kansasii* lung disease who did not receive antibiotic treatment compared with those who did (20% vs. 70%, *p* = 0.002). In accordance with the disease type, patients with *M*. *kansasii* lung disease who received antibiotic treatment had significantly higher proportion of cavitary lesions on chest radiographs and HRCT scans compared with patients who did not receive antibiotic treatment (*p* = 0.001 on chest radiographs and *p* < 0.001 on HRCT scans).

**Table 2:** Clinical manifestations of 54 patients diagnosed with *M. kansasii* lung disease.

	Total (n = 54, 100%)	Antibiotic treatment (n = 41, 76%)	Observation (n = 13, 24%)	*P* value
Males, n	39 (72)	31 (76)	8 (62)	0.478
Age, years	62 (45–66)	59 (45–66)	64 (53–74)	0.252
Body mass index, kg/m^2^	20.7 (18.7–22.8)	20.5 (18.7–22.4)	20.9 (18.3–25.4)	0.463
Symptom				
Cough	35 (65)	28 (68)	7 (54)	0.342
Sputum	40 (74)	31 (76)	9 (70)	0.722
Hemoptysis	11 (20)	10 (24)	1 (8)	0.261
Dyspnea	18 (33)	16 (39)	2 (15)	0.179
Positive AFB smear	36 (67)	30 (73)	6 (46)	0.096
Type of disease				
Fibrocavitary	24 (44)	23 (56)	1 (8)	0.002
Nodular- bronchiectatic	17 (32)	8 (20)	9 (70)	0.002
Unclassifiable	13 (24)	10 (24)	3 (23)	1.000
Chest radiograph findings				
Cavity	25 (46)	24 (59)	1 (8)	0.001
Bronchiectasis	23 (43)	15 (37)	8 (62)	0.113
Nodules or consolidation	30 (56)	23 (56)	7 (54)	0.887
HRCT findings				
Cavity	27 (50)	26 (63)	1 (8)	<0.001
Bronchiectasis	32 (59)	22 (54)	10 (77)	0.137
Centrilobular nodules	38 (70)	27 (66)	11 (85)	0.301
Consolidation	26 (48)	21 (51)	5 (39)	0.422

All data are presented as numbers (%) or as medians and interquartile ranges

AFB = acid-fast bacilli; HRCT = high-resolution computed tomography.

### Management and Treatment Outcomes in Patients With *M*. *kansasii* Lung Disease Who Received Antibiotic Treatment

Among the 41 patients who received antibiotic treatment, 16 (39%) initiated anti-tuberculosis treatment for presumed pulmonary tuberculosis until the definitive identification of *M*. *kansasii*. Regarding treatment regimens for *M*. *kansasii* lung disease, 29 (71%) patients received isoniazid, rifampin, and ethambutol, whereas 12 (29%) were treated with macrolide (azithromycin or clarithromycin), rifampin, and ethambutol (Table 3). The MIC of rifampin and clarithromycin via the broth microdilution method was available for 23 (56%) of the 41 patients who received treatment and 5 out of 23 patients had resistance to rifampicin.

**Table 3:** Management and treatment outcomes of the 41 patients who received antibiotic treatment for *M*. *kansasii* lung disease.

	No. of patients (%) or median (IQR)
Initially presumed as pulmonary tuberculosis	16 (39)
Treatment regimen for *M*. *kansasii* disease	
INH / RIF / EMB	29 (71)
Macrolide (AZT or CLR) / RIF / EMB	12 (29)
RIF susceptible, n/total n*	20 / 23 (87)
CLR susceptible, n/total n*	23 / 23 (100)
Treatment outcomes	
Completed treatment	24 (59)
Ongoing treatment	8 (20)
Lost or transferred during treatment	6 (15)
Discontinued treatment**	3 (7)
Death during treatment due to disease	0 (0)
Time from diagnosis to initiation of treatment, months	1.7 (0.3–3.8)
Time to sputum negative conversion, months	2.0 (1.0–3.0)
Treatment duration, months	15.0 (7.0–18.0)
Follow-up duration after diagnosis, months	24.1 (12.4–37.6)
Follow-up duration after treatment completion, months^†^	13.7 (2.7–45.2)

Data are presented as numbers (%) or as medians and interquartile ranges.

IQR = interquartile ranges; INH = isoniazid; RIF = rifampin; EMB = ethambutol; AZT = azithromycin; CLR = clarithromycin.

*The rifampin susceptibility test was performed in 23 patients.

**Two patients discontinued medications due to side effects like dyspepsia, anorexia, and urticarial; another patient self-discontinued due to improved respiratory symptoms.

^†^Calculated from the data of 24 patients who completed scheduled treatment.

All patients who completed the *M*. *kansasii* treatment course achieved symptomatic and radiographic improvement and negative microbiologic conversion. Relapse occurred in one patient after 5 months of following a successful 18-month treatment course of isoniazid, rifampin and ethambutol. This relapse was successfully treated with clarithromycin, rifampin and ethambutol for 12 months. Treatment of *M*. *kansasii* lung disease was discontinued in three (7%) patients due to dyspepsia, urticarial, and poor adherence. All patients who received antibiotic treatment for *M*. *kansasii* disease had negative microbiologic conversion after a median time of 2.0 months (IQR, 1.0–3.0 months). The median total treatment duration in 41 patients was 15.0 months (IQR, 7.0–18.0 months) and the median follow-up duration after completion of intended treatment in 24 patients was 13.7 months (IQR 2.7–45.2 months).

### Patients Who Were Not Diagnosed With *M*. *kansasii* Lung Disease

As shown in Table 4, among 50 patients who did not meet the 2007 ATS/IDSA criteria for *M*. *kansasii* lung disease, 27 (54%) already had another pulmonary disease, including other NTM lung disease due to MAC (n = 14), *Mycobacterium abscessus* complex (n = 1), or mixed infection with MAC and *M*. *abscessus* (n = 2) and pulmonary tuberculosis (n = 10). These 27 patients were followed for a median of 22.1 months (IQR, 10.6–27.7months) and a median 6.0 (IQR, 2.0–9.0) sputum cultures were obtained in follow-up period. The remaining 23 (46%) patients had only a single sputum isolate out of two or more respiratory specimens taken during the follow-up period; this result was insufficient to meet the microbiological criteria for NTM lung disease. Although these patients did not fulfill the 2007 ATS/IDSA diagnostic criteria, 5 out of the 23 (22%) were treated with a presumed diagnosis of pulmonary tuberculosis or *M*. *kansasii* lung disease. The remaining 18 of 23 (78%) patients were observed without antibiotic treatment for a median 12.1 months (IQR, 4.4–32.0 months) follow-up period. A median of 4.0 (IQR 2.8–10.3) sputum cultures were performed and none of these patients developed *M*. *kansasii* lung disease.

**Table 4:** Reasons why 50 patients did not meet the 2007 ATS/IDSA diagnostic criteria for *M*. *kansasii* disease.

Reason	No. of patients (%)
**Clinical criteria**	
Excluded due to other disease	27 (54)
*Mycobacterium avium* complex	14
*Mycobacterium abscessus* complex	1
Mixed NTM infection*	2
Pulmonary tuberculosis**	10
**Microbiologic criteria**	
Culture-positive from only a single sputum sample^†^	23 (46)

*Two patients were diagnosed with mixed infection (*M*. *avium* complex and *M*. *abscessus*).

**Pulmonary tuberculosis was demonstrated by the presence of an *M*. *tuberculosis* culture isolate or by polymerase chain reaction.

^†^Of these patients, 18 (78%) underwent follow-up without antibiotic treatment for a median duration of 12.1 months (interquartile range 4.4–32.0 months). None of these patients developed *M*. *kansasii* lung disease.

## Discussion

This study evaluated the clinical relevance and disease characteristics in 104 patients from whom M. *kansasii* respiratory isolates were obtained. We found that 52% of all patients with *M*. *kansasii*-positive respiratory cultures were ultimately diagnosed with *M*. *kansasii* lung disease; 76% of these patients received antibiotic treatment and had a favorable outcome. Moreover, *M*. *kansasii* lung disease exhibited diverse radiographic findings, including the nodular bronchiectatic form. Finally, we found that the isolation of *M*. *kansasii* from patients with tuberculosis or another NTM disease or a single sputum isolate was not associated with the development of *M*. *kansasii* lung disease during the follow-up period in patients who did not satisfy the diagnostic criteria.

Our study included more than 100 patients, none of whom had HIV. Moreover, 52% of all patients from whom an *M*. *kansasii* respiratory isolate was obtained had clinically relevant disease. This finding is consistent with recent epidemiologic studies in Israel, Croatia and the Netherlands using the 2007 ATS/IDSA diagnostic criteria [25–27]. However, the clinical significance of *M*. *kansasii* respiratory isolates has been shown to vary from 17% to 88% (Table 5). This wide range of clinical significance might be explained by small study sizes [28–30], the inclusion of many patients with pneumoconiosis [9] or HIV [11, 31], or the use of previous diagnostic criteria [10, 12, 32]. One epidemiologic study in the UK [33] excluded patients with a single sputum isolate, leading to a relatively high estimation of the clinical relevance of *M*. *kansasii* respiratory cultures.

**Table 5:** Clinical relevance of *M*. *kansasii* respiratory isolates in previous reports.

Author, year, reference	Country	Study period	Clinical relevance*
Fogan, 1969 [34]	Oklahoma, USA	1966–1968	50% (18/36)
Jenkins, 1981 [9]	Wales, UK	1952–1978	84% (154/184)
O’Brien, 1987 [10]	USA	1981–1983	75% (762/1016)
Pang, 1991 [35]	Australia	1962–1987	48% (39/81)
Debrunner, 1992 [32]	Switzerland	1983–1988	26% (9/35)
Bloch, 1998 [11]	California, USA	1992–1996	88% (236/270)**
Corbett, 1999 [31]	South Africa	1996–1997	41% (23/56)^†^
Koh, 2006 [16]	South Korea	2002–2003	50% (7/14)
Bodle, 2008 [28]	New York City, USA	2000–2003	70% (7/10)
Van Ingen, 2009 [29]	Netherlands	1999–2005	71% (12/17)
Thomson, 2010 [36]	Australia	2005	53% (10/19)
Winthrop, 2010 [30]	Oregon, USA	2005–2006	38% (3/8)
Simons, 2011 [12]	Asia	1971–2007	17% (34/198)
Davies, 2012 [33]	UK	2000–2007	73% (40/55)
Braun, 2012 [25]	Israel	2004–2010	50% (7/14)
Jankovic, 2013 [26]	Croatia	2006–2010	50% (5/10)
Chien, 2014 [37]	Taiwan	2000–2012	44% (234/526)
Gommans, 2015 [27]	Netherlands	2001–2011	53% (10/19)
Current study	South Korea	2003–2014	52% (54/104)

USA = United States of America, UK = United Kingdom.

*Proportion of patients judged to have *M*. *kansasii* lung disease out of all patients from whom *M*. *kansasii* had been isolated.

**187 (69%) were HIV-positive.

^†^40 (34%) were HIV-positive.

In contrast to previous findings regarding the virulence and clinical significance of a single *M*. *kansasii* isolate, we found that 18 patients with a single sputum isolate did not develop *M*. *kansasii* lung disease during the follow-up period. This result supports the 2007 ATS/IDSA diagnostic criteria for infection with *M*. *kansasii*, MAC, and *M*. *abscessus* complex, according to which a single positive sputum culture is regarded as indeterminate for the diagnosis of NTM lung disease [5].

Patients with *M*. *kansasii* lung disease typically present with upper-lobe fibronodular infiltrates with cavitation on chest radiographs [7]. In our study, the nodular bronchiectatic and unclassifiable forms were also commonly observed in patients with *M*. *kansasii* lung disease, especially those who did not receive antibiotic treatment. This finding is consistent with a recent study [27] in which, various radiographic findings of *M*. *kansasii* lung disease were reported. Our findings imply that patients with *M*. *kansasii* lung disease can also exhibit the nodular bronchiectatic form, similar to other patients with NTM lung disease.

Regarding treatment for *M*. *kansasii* lung disease, we found that 76% of all patients with *M*. *kansasii* disease received antibiotic treatment, while the remaining 24% presented an indolent course and did not require antibiotic treatment. Similar to previous reports of good prognoses [5, 7, 38], all patients who completed treatment showed a favorable response. Moreover, no treatment failure occurred, despite the presence of rifampin-resistant isolates in 3 out of the 23 patients. Regarding treatment regimen, 29% of all patients who underwent antibiotic treatment for *M*. *kansasii* lung disease received a macrolide-containing regimen with rifampin. The current ATS/IDSA recommendation for treating *M*. *kansasii* lung disease is an 18-month regimen consisting of daily isoniazid, rifampin, and ethambutol therapy. However, untreated *M*. *kansasii* strains are easily inhibited *in vitro* by clarithromycin at lower concentrations than those required for MAC treatment [39], a result that is readily achievable with the standard therapeutic doses. In addition, a preliminary study showed that the administration of clarithromycin, rifampin, and ethambutol for the treatment of *M*. *kansasii* lung disease resulted in favorable treatment outcomes in 18 patients [40]. At our institution, the treatment protocol was changed from including isoniazid to including macrolide antibiotics in January 2013 after discussion regarding treatment of *M*. *kansasii* lung disease. Although our sample size was small and some patients are still treatment, our study yielded important preliminary observations regarding the potential of regimens containing macrolide, rifampin, and ethambutol for treating *M*. *kansasii* lung disease. Further studies with larger sample sizes are needed to confirm this finding.

Our study did have several limitations. First, this retrospective study was conducted at a single referral institution and thus we were unable to calculate the population-based prevalence. Second, the clinical significance of *M*. *kansasii* positivity in patients with *M*. *kansasii* isolates who were administered antibiotics (including rifampin) due to accompanying tuberculosis or another NTM disease remains unclear. In addition, as genotyping was not performed in one recurred case, relapse could not be distinguished from re-infection. Finally, although *M*. *kansasii* is a heterogeneous species with several distinct subtypes [41], clinical significance was not evaluated according to *M*. *kansasii* subtype.

In conclusion, about half of all patients from whom *M*. *kansasii* was isolated from the respiratory specimen presented clinically significant lung disease with diverse radiographic findings. Similar to other NTM lung disease, patients with *M*. *kansasii* lung disease can exhibit the nodular bronchiectatic form, especially with an indolent clinical course. The clinical significance of a single *M*. *kansasii* isolate was not conclusive, suggesting strict adherence to the recommended diagnostic criteria.
